# Discontinuation of Immunotherapy in Patients With Relapsing Myelitis Without AQP4/MOG Antibodies

**DOI:** 10.1002/acn3.70063

**Published:** 2025-05-02

**Authors:** Ki Hoon Kim, You‐Ri Kang, Jae‐Won Hyun, Su‐Hyun Kim, Ho Jin Kim

**Affiliations:** ^1^ Department of Neurology Severance Hospital, Yonsei University College of Medicine Seoul Korea; ^2^ Department of Neurology Research Institute and Hospital of National Cancer Center Goyang Korea; ^3^ Department of Neurology Chonnam National University Hospital Gwangju Korea

**Keywords:** discontinuation, immunotherapy, myelitis

## Abstract

This study assesses the outcomes of immunotherapy discontinuation in patients with relapsing seronegative idiopathic myelitis (SIM), a condition that remains uninvestigated due to its rarity. We reviewed records from 77 patients with relapsing SIM at the National Cancer Center of Korea, focusing on 11 who discontinued treatment after a median relapse‐free period of over 5 years. Notably, all patients remained relapse‐free for a median of 36.5 months following discontinuation. Our findings suggest that immunotherapy discontinuation can be a feasible therapeutic strategy for selected patients who have maintained a prolonged relapse‐free period.

## 1 Introduction

Seronegative idiopathic myelitis (SIM), a diverse range of inflammatory conditions affecting the spinal cord, presents significant challenges in its management due to the absence of definitive serological markers and heterogeneous clinical manifestations [1, 2]. Unlike more defined conditions with specific antibodies such as neuromyelitis optica spectrum disorder (NMOSD) and myelin oligodendrocyte glycoprotein‐antibody‐associated disease (MOGAD) [3, 4], SIM requires an individual therapeutic approach due to the paucity of solid evidence from dedicated studies.

While no standardized treatment protocols exist, long‐term immunotherapy is often used for managing relapsing form of SIM (relapsing SIM) to control inflammatory activity [5]. However, concrete data about long‐term therapeutic strategy, including the optimal duration of immunotherapies, remains sparse, leaving clinicians to rely on limited experiences or extrapolations from similar cases.

In this study, we aimed to assess the outcomes following the cessation of long‐term immunotherapies in patients with relapsing SIM, thereby offering more evidence‐based treatment strategies.

## 2 Methods

**FIGURE 1:**
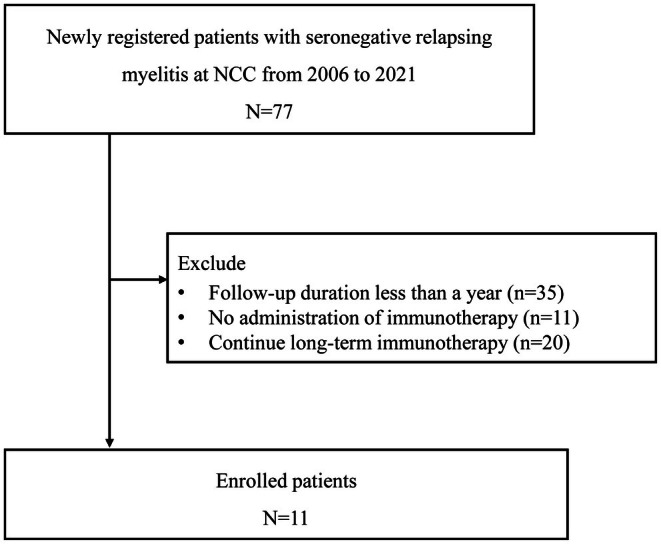
A flow diagram of patients. During the study period, 77 patients were diagnosed with relapsing SIM, and 11 patients who fulfilled the inclusion criteria were selected. NCC National Cancer Center of Korea; SIM, seronegative idiopathic myelitis.

This retrospective study reviewed the medical records of 77 patients diagnosed with the relapsing SIM at the National Cancer Center of Korea (NCC) from 2007 to 2021. All patients presented with sensory, motor, or autonomic deficits (one or any combination) attributed to the spinal cord, which develop over hours to days (nadir ≤ 21 days) [1]. A diagnosis of inflammatory myelitis is supported by MRI findings of T2 hyperintense signal in the spinal cord with or without gadolinium enhancement, CSF pleocytosis, and the exclusion of vascular, compressive, cancer‐related, infectious, and metabolic causes. The initial comprehensive workup at the first visit included hematological, biochemical assays, and autoimmune screenings such as antinuclear antibody, antineutrophil cytoplasmic antibody, and anti‐Ro/La antibody to exclude systemic causes of myelitis. It also encompassed testing for specific infections like varicella zoster virus, human immunodeficiency virus, measles, hepatitis B and C, and tuberculosis. During the follow‐up period, blood samples were obtained at least once at the clinic, and all patients tested negative for anti‐aquaporin‐4 (AQP4) and anti‐myelin oligodendrocyte glycoprotein (MOG) antibodies using a live cell‐based assay [6, 7]. For cases who had visited before these assays were established, stored samples were tested later. In addition, a serologic examination was performed at relapses in cases where serum samples were available. Among the patients with SIM, eligible patients met the following inclusion criteria: (1) a history of at least two acute episodes of myelitis (nadir ≤ 21 days) without any evidence of brain involvement, (2) a regular follow‐up with 2–6 months interval over 1 year, during which long‐term immunotherapies (> 12 months) except oral methylprednisolone were administered, and (3) discontinuation of immunotherapies during the follow‐up period. Figure 1 provides a flow diagram illustrating the inclusion of the 11 patients who ultimately met study criteria. Patients were selected from the NCC registry for CNS demyelinating disease, and this study protocol was approved by the Institutional Review Board of the NCC.

## 3 Results

Table 1 summarizes the demographics and clinical findings of the enrolled patients. The median age at first myelitis episode was 51 years (range: 27–61 years), and the male:female ratio was 8:3. The median duration of immunotherapy was 62.3 months (interquartile range [IQR]: 52.2–68.0), and the median expanded disability status scale score was 2.5 (IQR: 2.0–2.8) at the time of immunotherapy initiation. Eight (73%) patients had experienced a longitudinally extensive transverse myelitis (LETM) at least once. Mycophenolate mofetil was the most administered drug (*n* = 7), followed by azathioprine (*n* = 2). Before their referral to our institution, two patients were treated with disease‐modifying therapy (DMT) for multiple sclerosis (MS), one on dimethyl fumarate and the other on interferon‐beta; after a thorough evaluation, MS was excluded for both, leading to discontinuation of their DMT. Three patients (27%) experienced further myelitis episodes under immunotherapy. The median annual relapse rate was reduced from 2.0 (IQR: 1.0–2.7) before immunotherapy to 0 (IQR: 0.0–0.1) during immunotherapy.

**TABLE 1:** Demographics and clinical findings of enrolled patients.

Patients (*n* = 11)	
Sex, male	8 (73%)
Age at first myelitis episode, years, median [range]	51 [27–61]
Time from disease onset to immunotherapy, years, median (IQR)	1.5 (0.9–2.6)
Number of myelitis episodes before immunotherapy, median (IQR)	3 (2–3)
Patients with LTEM	8 (73%)
ARR before immunotherapy, median (IQR)	2.0 (1.0–2.7)
EDSS at immunotherapy initiation, median (IQR)	2.5 (2.0–2.8)
Type of immunotherapy, *n* (%)	
Azathioprine	2 (18%)
Mycophenolate mofetil	7 (63%)
Mitoxantrone	1 (9%)
Disease modifying therapy for multiple sclerosis	2 (18%)
Maintenance duration of immunotherapy, month, median (IQR)	62.3 (52.2–68.0)
Patients experiencing myelitis episodes under immunotherapies, *n* (%)	3 (27%)
ARR during immunotherapy, median (IQR)	0 (0.0–0.1)
EDSS at immunotherapy discontinuation, median (IQR)	2.0 (2.0–2.0)
Follow‐up period after immunotherapy discontinuation, months, median [range]	36.5 [12.1–94.5]
Clinical relapses after immunotherapy discontinuation, *n*	0

Abbreviations: ARR, annual relapse rate; EDSS, expanded disability status scale; LETM, longitudinally extensive transverse myelitis.

**FIGURE 2:**
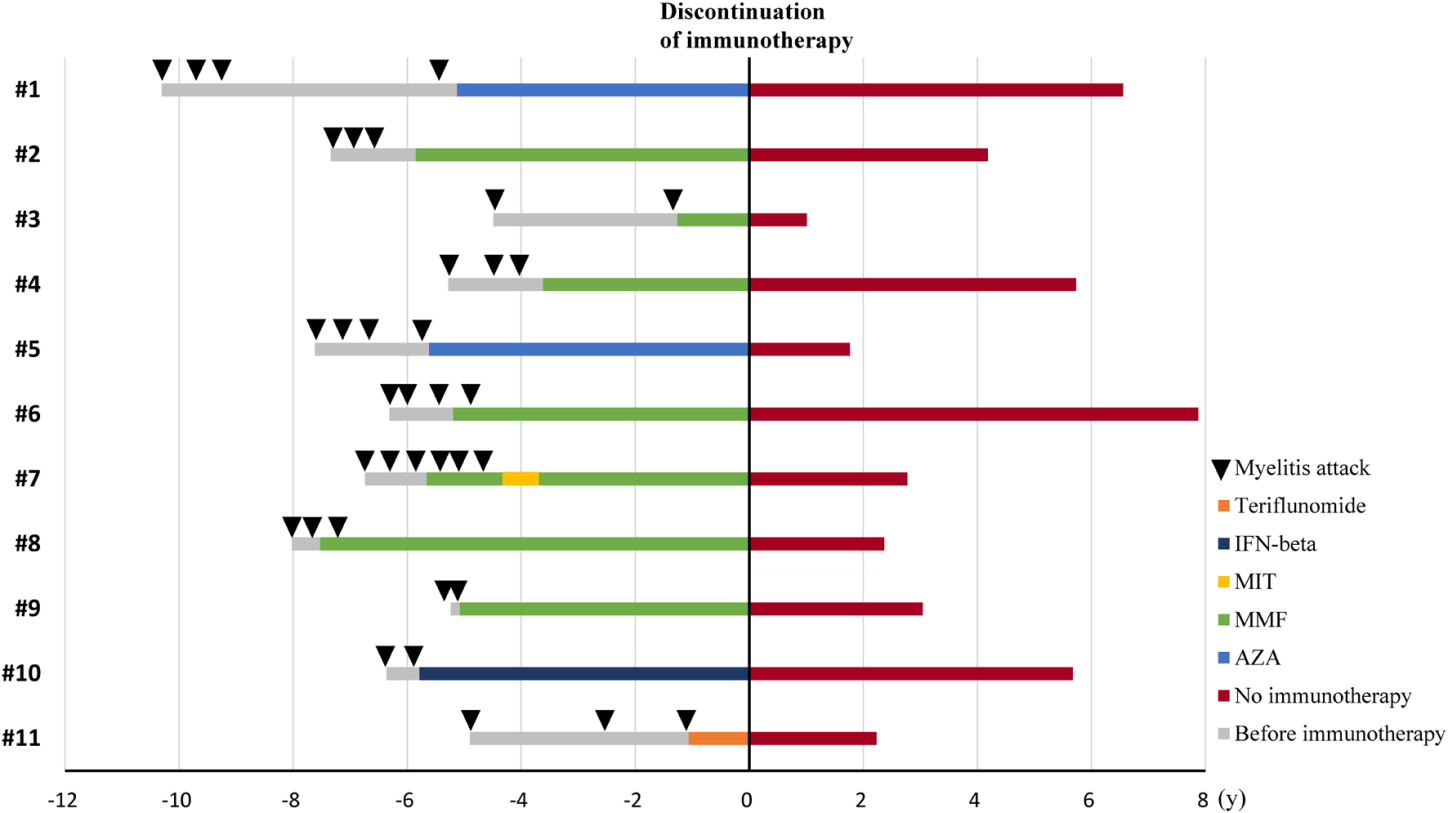
Disease course and immunotherapies of enrolled patients. Black arrows indicate myelitis attacks, and the horizontal axis indicates the time of immunotherapy discontinuation. AZA, azathioprine; IFN‐beta, interferon‐beta; MIT, mitoxantrone; MMF, mycophenolate mofetil.

The median relapse‐free period from the last myelitis episode to cessation of immunotherapy was 61.5 months (IQR: 51.9–68.4). Reasons for immunotherapy discontinuation included the prolonged periods without further relapses (*n* = 9), side effects of immunotherapy (*n* = 1), and preparing for pregnancy (*n* = 1). Following the discontinuation of immunotherapies, all patients remained relapse‐free for a median follow‐up duration of 36.5 months (range: 12.1–94.5), without reported disability worsening. The disease course and immunotherapies of enrolled patients are depicted in Figure 2. In addition, detailed clinical and MRI information of each patient is provided in Table S1.

## 4 Discussion

In our cohort of patients with relapsing SIM, those who had maintained a relapse‐free status under immunotherapy over a median of 5 years showed no further relapses more than 36 months after discontinuing the treatment. These findings indicate that immunotherapy discontinuation can be a feasible therapeutic strategy for this type of patient, provided a substantial long period without relapses is achieved.

In contrast, the likelihood of a further relapse associated with discontinuation or de‐escalation of immunotherapy remains high in AQP4‐positive NMOSD. Previously, we have reported that 82% of patients relapsed after a median of 6 months of immunotherapy discontinuation in patients with AQP4‐positive NMOSD [8]. In addition, Demuth et al. reported that de‐escalation of rituximab therapy is associated with an increased risk of disease reactivation, regardless of AQP4 positivity [9]. Thus, patients with NMOSD typically continue immunosuppressive treatments indefinitely due to the high risk of severe relapses, which can lead to devastating disability. In MS, the decision to discontinue DMTs is complex and involves careful consideration of various factors, including age, disease duration, and stability [10, 11]. Although the cessation of immunotherapy in older patients with MS or other CNS demyelinating diseases is considered due to concerns about increased infectious complications [12, 13], limited patients with MS may be candidates for discontinuing DMTs under specific circumstances. The favorable result in our relapsing SIM cohort after stopping immunotherapy suggests a possible divergence in the underlying disease pathophysiology when compared with NMOSD and MS. In relapsing SIM, prolonged immunomodulation may induce lasting immunological changes that continue beyond the treatment period, potentially reconfiguring the immune system to prevent future disease activity.

This study is limited by its retrospective design and small sample size. Additionally, it was confined to Asian patients from a single center, which may restrict the generalizability of the results. Future multicenter studies including other ethnic groups are essential to validate our results. Finally, the decision to discontinue immunotherapy was made on a case‐by‐case basis in consultation with each patient, potentially leading to a selection bias toward those with less disability. Despite these constraints, it represents the first exploration of the feasibility of discontinuing immunotherapy in patients with relapsing SIM.

## Author Contributions

Conceptualization: Ki Hoon Kim and Ho Jin Kim. Resources: All authors. Supervision: Ho Jin Kim. Visualization: Ki Hoon Kim. Writing – original draft: Ki Hoon Kim. Writing – review and editing: All authors.

## Conflicts of Interest

Y.‐R. Kang report no disclosures. K.H. Kim has received a grant from the Korean Society of Neuroimmunology. S.‐H. Kim has lectured, consulted, and received honoraria from Bayer Schering Pharma, Biogen, Genzyme, Merck Serono, and UCB and received a grant from the National Research Foundation of Korea. J.‐W. Hyun has received a grant from the National Cancer Center in Korea. H.J. Kim received a grant from the National Research Foundation of Korea and research support from AprilBio, Eisai, Good T cells, and UCB; received consultancy/speaker fees from Alexion, Altos Biologics, AstraZeneca, Biogen, Daewoong Pharmaceutical, Eisai, GC Pharma, Handok Pharmaceutical, Kaigene, Kolon Life Science, MDimune, Merck, Mitsubishi Tanabe Pharma, Roche, and Sanofi; is a coeditor for the Multiple Sclerosis Journal and an associate editor for the Journal of Clinical Neurology.

## Supporting information


**Table S1.** Detailed clinical and MRI findings of enrolled patients.

## Data Availability

The anonymized datasets used in this study are available from the corresponding author on reasonable request.
