# Supplementary material for: Discontinuation of Immunotherapy in Patients With Relapsing Myelitis Without AQP4/MOG Antibodies
Source: Ann Clin Transl Neurol. 2025 May 2;12(8):1689–92. doi: 10.1002/acn3.70063 (PMC12343319; doi:10.1002/acn3.70063)
Supplement: Supplementary file 1 — Table S1. Detailed clinical and MRI findings of enrolled patients. [file ACN3-12-1689-s001.docx]

Supplementary Table 1. Detailed clinical and MRI findings of enrolled patients.

|  | Case 1 | Case 2 | Case 3 | Case 4 | Case 5 | Case 6 | Case 7 | Case 8 | Case 9 | Case 10 | Case 11 |
| --- | --- | --- | --- | --- | --- | --- | --- | --- | --- | --- | --- |
| Sex | Male | Male | Female | Male | Male | Male | Male | Female | Male | Male | Female |
| Age at onset, years | 28 | 46 | 54 | 50 | 56 | 51 | 52 | 60 | 61 | 48 | 27 |
| Underlying disease | None | HTN, Cerebellar infarction | None | HTN, dyslipidemia | DM | None | None | Osteoporosis, dyslipidemia | DM, dyslipidemia | None | Thyroid cancer |
| Brain MRI finding | Normal | Ischemic lesion at left cerebellum | Normal | Not done | Normal | Several T2 spotty high lesions in deep white matter | Several T2 spotty high lesions in deep white matter | Several T2 spotty high lesions in deep white matter | Several T2 spotty high lesions in deep white matter | Normal | Normal |
| Spine MRI finding  (T2 hyperintense extent) | T5-11/ T8-9/ T8 | C1-4/ C1-3 / medulla-C3 | C2-5/ Conus | T8-9/ T8-10/ T9-10 | C7-8/ C7-T2/ C7 | T7-8/ T6-9/ T7 | T6-8/ T7-11/ T4-11 | C5-T6/ T1-2 | Medulla-C1/ C3 | T3/ T3-4 | C2-3/ C2-7/ C7 |
| CSF finding at first myelitis episode | R0 W0 Protein 23 OCB negative | R1 W0 Protein 42 | R0 W40 Protein 49 OCB negative | R0 W0 Protein 26 OCB negative | Not done | R0 W6 Protein 48 OCB positive | R0 W0 Protein 37 OCB positive | R0 W0 Protein 30 OCB negative | R0 W6 Protein 47 OCB negative | Not done | R150 W12 Protein 36 OCB negative |
| Time interval from onset to treatment, years | 5.2 | 1.5 | 3.2 | 1.7 | 2.0 | 1.1 | 1.1 | 0.5 | 0.2 | 0.6 | 3.8 |
| Number of myelitis episodes before treatment | 4 | 3 | 2 | 3 | 4 | 3 | 3 | 2 | 2 | 2 | 3 |
| ARR before immunotherapy | 0.77 | 2.02 | 0.62 | 1.81 | 2.00 | 2.68 | 2.77 | 4.03 | - (< 6months) | 3.46 | 0.78 |
| EDSS at treatment initiation | 3.5 | 2.5 | 2.0 | 2.5 | 2.5 | 2.0 | 3.0 | 3.0 | 2.0 | 2.5 | 2.0 |
| Type of immunotherapies | Azathioprine | MMF | MMF | MMF | Azathioprine | MMF | MMF, Mitoxantrone | MMF | MMF | Beta-interferon | Teriflunomide |
| Treatment duration, months | 61.5 | 70.2 | 15.1 | 43.4 | 67.4 | 62.3 | 67.9 | 90.3 | 60.9 | 68.1 | 12.7 |
| Attacks during treatment [involvement location] | 0 | 0 | 0 | 0 | 0 | 1, [T9-11] | 4, [T4-11, T8-9, Conus, T10-11] | 1, [T1-6] | 0 | 0 | 0 |
| ARR during immunotherapy | 0.0 | 0.0 | 0.0 | 0.0 | 0.0 | 0.19 | 0.71 | 0.13 | 0 | 0 | 0 |
| EDSS at discontinuation | 2.0 | 2.0 | 2.0 | 2.0 | 2.0 | 3.5 | 4.5 | 2.0 | 0 | 2.0 | 1.0 |
| Reason for discontinuation | Relapse-free over several years | Relapse-free over several years | Drug side effect (diarrhea) | Relapse-free over several years | Relapse-free over several years | Relapse-free over several years | Relapse-free over several years | Relapse-free over several years | Relapse-free over several years | Relapse-free over several years | Plan for pregnancy |
| Follow-up after treatment discontinuation, months | 78.7 | 50.2 | 12.1 | 68.8 | 21.2 | 94.5 | 33.3 | 28.4 | 36.5 | 68.1 | 26.8 |
| Myelitis episodes after treatment discontinuation | 0 | 0 | 0 | 0 | 0 | 0 | 0 | 0 | 0 | 0 | 0 |

Abbreviations: OCB, oligoclonal band; EDSS, expanded disability status scale; ARR, annual relapse rate; MMF, mycophenolate mofetil
